# Multi-Gene Analysis Reveals a Lack of Genetic Divergence between *Calanus agulhensis* and *C. sinicus* (Copepoda; Calanoida)

**DOI:** 10.1371/journal.pone.0045710

**Published:** 2012-10-31

**Authors:** Robert Kozol, Leocadio Blanco-Bercial, Ann Bucklin

**Affiliations:** Department of Marine Sciences, University of Connecticut - Avery Point, Groton, Connecticut, United States of America; Instituto de Higiene e Medicina Tropical, Portugal

## Abstract

The discrimination and taxonomic identification of marine species continues to pose a challenge despite the growing number of diagnostic metrics and approaches. This study examined the genetic relationship between two sibling species of the genus *Calanus* (Crustacea; Copepoda; Calanidae), *C. agulhensis* and *C. sinicus*, using a multi-gene analysis. DNA sequences were determined for portions of the mitochondrial cytochrome *c* oxidase I (mtCOI); nuclear citrate synthase (CS), and large subunit (28S) rRNA genes for specimens collected from the Sea of Japan and North East (NE) Pacific Ocean for *C. sinicus* and from the Benguela Current and Agulhas Bank, off South Africa, for *C. agulhensis*. For mtCOI, *C. sinicus* and *C. agulhensis* showed similar levels of haplotype diversity (H_d_ = 0.695 and 0.660, respectively) and nucleotide diversity (π = 0.003 and 0.002, respectively). Pairwise F_ST_ distances for mtCOI were significant only between *C. agulhensis* collected from the Agulhas and two *C. sinicus* populations: the Sea of Japan (F_ST_ = 0.152, p<0.01) and NE Pacific (F_ST_ = 0.228, p<0.005). Between the species, F_ST_ distances were low for both mtCOI (F_ST_ = 0.083, p = 0.003) and CS (F_ST_ = 0.050, p = 0.021). Large subunit (28S) rRNA showed no variation between the species. Our results provide evidence of the lack of genetic distinction of *C. sinicus* and *C. agulhensis*, raise questions of whether *C. agulhensis* warrants status as a distinct species, and indicate the clear need for more intensive and extensive ecological and genetic analysis.

## Introduction

The taxonomic relationships of closely related species provide vital information for accurate assessment and conservation of marine biodiversity. However, identifying diagnostic characteristics for species identification and agreeing on exact delimitation of species boundaries has remained challenging. Molecular phylogenetic analysis provides a reliable and independent means to evaluate evolutionary and taxonomic relationships and examine species boundaries among closely related and cryptic species [1], [2], [3]. Molecular systematic and phylogenetic studies of marine zooplankton have resulted in the revision of many pelagic marine taxa [4], [5], including copepods [6], [7], [8], [9].

The planktonic marine copepod family, Calanidae (Crustacea: Copepoda: Calanoida) includes eight genera and 29 species that share highly similar morphological characteristics and overlapping species' distributions [10]. Evolutionary relationships within the Calanidae continue to be a topic of debate [11]. The genus *Calanus* comprises 14 species, including 11 that have been assorted into two sibling species groups: the finmarchicus group (*C. finmarchicus*, *C. glacialis*, *C. marshallae*) and the helgolandicus group (*C. helgolandicus*, *C. agulhensis*, *C. australis*, *C. chilensis*, *C. euxinus*, *C. jashnovi*, *C. pacificus*, *C. sinicus*), as well as three ungrouped species (*C. hyperboreus*, *C. simillimus* and *C. propinquus*; [12], [10]). The sibling species are discriminated in many cases by very subtle morphological and morphometric characters, primarily secondary sexual characters [13], [14], and species identifications are frequently based on individual size and geographical collection location.

### Taxonomy and Ecology of the Species

*Calanus agulhensis* was first documented by Cleve [15] as *C. finmarchicus* off the coast of South Africa. A distinct new species was described by De Decker et al. [16] based on subtle morphological characters and geographical isolation of the South African populations from those of *C. australis* and *C. pacificus*. In particular, De Decker et al. [16] differentiated *C. agulhensis* from *C. australis* by physical characteristics such as shorter first antennae of the females and detailed structures of the fifth thoracic leg of both males and females. De Decker et al. [16] gave the type locality as the Agulhas Bank, off the southern tip of South Africa, which he considered to be the center of distribution, where the species was observed to spawn year-round, with decreased abundance to the west. The species is also found off the east and west coasts of South Africa, with relatively high abundance off the western shelf from November through December [17]. Within this region, *C. agulhensis* dominates the zooplankton community, comprising up to 85% of copepod biomass on the western bank [18].

*Calanus sinicus* was first described by Brodsky [19], although lack of a type locality and type specimen caused it to become a *nomen nudum*. A new diagnostic of the species was made with the type locality identified as Tsindao, Yellow Sea [20]. The species distribution includes the South and East China Sea, Yellow Sea, Bohai Sea and the Sea of Japan [20], [21], [22]. Reproduction of *C. sinicus* occurs year-round in the Sea of Japan, reaching a maximum reproductive rate between June and August depending on the region [23], [24].

Comparison of descriptions made by Hulsemann [20] and DeDecker et al. [16] reveals similarities in several morphological features. Adult females share similar averages in body length, 2.73 mm for *C. agulhensis* and 2.95 mm for *C. sinicus*. The first antenna for *C. agulhensis* reaches beyond the furcal rami by one segment, while *C. sinicus* reaches beyond the furcal rami by one or two segments. Genital segments for both are described as being as long as they are broad. Both species average 18 teeth on the inner segment of leg V for adult males. The heads also show similarities such as an anterior bulge dorsal of the rostral attachments. Other comparisons were difficult to make because of a lack of descriptive standards and physical analyses. DeDecker et al. [16] chose to do a full description of both the males and females, however leaving out pore signatures. Hulsemann [20] chose to analyze differences between *C. sinicus* and *C. joshnovi* to give recognition to *C. sinicus* as a species, leaving out a full description and focusing largely on pore signatures. Additional morphological comparisons may be required (B.W. Frost, Univ. Washington, pers. comm.).

The sibling species *C. agulhensis* and *C. sinicus* are continental shelf species that prosper in mid-shelf ecosystems [9]. These areas are characterized by moderate temperatures, with optimal food supply and water depth. Both species are integral members of the zooplankton because of their large size, abundance and role as secondary producers for important commercial fish species [23], [25]. *Calanus agulhensis* is a major prey species for anchovy, pilchard, herring, hake and horse mackerel; it is estimated that the species makes up to 60% of the diet of round herring, while *C. sinicus* is a major prey species for anchovies, sardine, and sand eels [22].

The coastal ocean regions surrounding South Africa and Japan have similarly dynamic hydrography. The Benguela Current and western Agulhas Bank regions are characterized by seasonal winds and current-driven coastal upwelling [26], [27]. The North Pacific western boundary current influences coastal areas to the east of Japan: the Kii Channel and Sagami Bay exhibit estuarine upwelling and micro-scale upwelling plumes, respectively [22], [27]–[29]. These dynamic physical processes throughout the species' ranges highlight their shared ability to resist advection and establish populations in the most advantageous regions [9], [19].

### Molecular systematic analysis

The taxonomic and systematic relationships among *Calanus* species have been examined using molecular characters [9], [30]–[33]. Dates of evolutionary divergence among the species, once considered to be on the order of tens or hundreds of thousands of years [13], [34], have been estimated to be on the order of tens of millions of years [31].

Despite the morphological similarity, overlapping ranges, and circumglobal distributions of many species, DNA sequence variation of diverse gene regions has been shown to correctly identify and discriminate species of copepods [7], [35]–[39], [40], including most species of *Calanus* [30]–[32], [41], [42]. An exception is the lack of significant divergence between *C. helgolandicus* and *C. euxinus*, which has called into question their status as distinct species [33], [43].

Analysis of multiple gene regions is critical for accurate resolution of species relationships; the selection of markers with diverse evolutionary drivers is particularly important [41], [44]–[46]. In this study, we analyzed the taxonomic relationship between two sibling species, *C. agulhensis* and *C. sinicus*, based on DNA sequences for portions of three genes: mitochondrial cytochrome *c* oxidase subunit I (mtCOI), citrate synthase (CS), and nuclear large subunit (28S) rRNA. MtCOI – especially the so-called “barcode region” [47] – exhibits useful patterns of inter- and intraspecific variation for diagnostic analysis of evolutionary relationships among congeneric species of many metazoans [48], [49], including calanoid copepods [41]. MtCOI has also been used to examine population genetic structure of several species of *Calanus*, including: *C. helgolandicus* [33] *C. pacificus* [50], and *C. sinicus* [51]. The nuclear gene CS was selected to provide a diploid marker for better resolution of breeding patterns, including possible interbreeding and hybridization, among the closely-related species. CS has also been used to discern significant intraspecific variation of *C. finmarchicus* in the N. Atlantic Ocean [46]. Although it is less reliable as a diagnostic tool at the species level, the slowly-evolving 28S rRNA gene was chosen to better resolve the deeper branches between selected species of the two sibling species groups of *Calanus*. This gene has previously been used as a reliable comparative and “support” gene for mtCOI analyses [52], [53]. The combined use of DNA sequences for mitochondrial and nuclear protein-coding genes and a nuclear rRNA gene provides us with a broad genetic spectrum for analysis of evolutionary and taxonomic relationships among species of this challenging copepod genus.

## Methods

### Sample Collection and Processing

**Figure 1:**
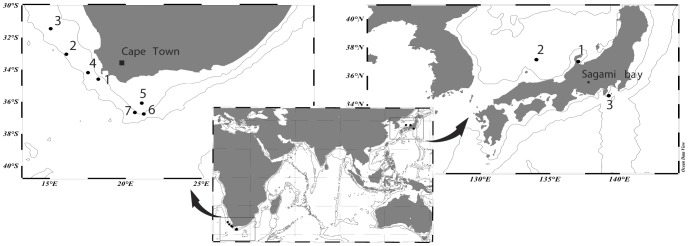
Geographic locations of the samples analyzed for this study. Collections of *C. sinicus* were made in three locations to the West and East of Japan; *C. agulhensis* was collected to the south and west of South Africa. Numbers correspond to stations listed in Table 1. Populations are represented by stations 1, 2, 3 and 4 for Benguela and stations 5, 6 and 7 for Agulhas (left panel); stations 1 and 2 for West Japan and station 3 for East Japan (right panel).

Samples containing *C. sinicus* were collected from two stations to the west of Japan, in the Sea of Japan, and one station to the east, in the NE Pacific Ocean. Samples of *C. agulhensis* were collected from seven stations to the south and west of South Africa, including four stations to the west in the Benguela Current System and three stations from the Agulhas Bank (Fig. 1; Table 1 and 2). Samples were preserved in 95% ethanol and stored at 4°C.

**Table 1:** Collection information for samples used in the multi-gene analysis.

Species	Station	Population	Location	Collection Date
*C. agulhensis*	1	Benguela	34.302 S, 18.079 E	12/14/2005
*C. agulhensis*	2	Benguela	33.038 S, 16.082 E	3/21/2000
*C. agulhensis*	3	Benguela	31.447 S, 15.054 E	3/22/2003
*C. agulhensis*	4	Benguela	34.218 S, 17.835 E	12/14/2005
*C. agulhensis*	5	Agulhas	36.085 S, 21.052 E	11/14/2001
*C. agulhensis*	6	Agulhas	36.761 S, 21.186 E	11/14/2001
*C. agulhensis*	7	Agulhas	36.669 S, 20.597 E	11/9/2001
*C. sinicus*	1	West Japan	37.000 N, 137.014 W	6/22/2001
*C. sinicus*	2	West Japan	37.140 N, 133.000 W	6/20/2001
*C. sinicus*	3	East Japan	34.599 N, 139.200 W	3/13/2010

Population names are as used in the text and statistical analyses.

**Table 2:** Numbers of individuals sequenced and analyzed per sampling station (N).

Species	Station	Population	mtCOI (N)	CS (N)	28S (N)
*C. agulhensis*	1	Benguela	0	0	1
*C. agulhensis*	2	Benguela	13	12	1
*C. agulhensis*	3	Benguela	11	6	2
*C. agulhensis*	4	Benguela	0	1	2
*C. agulhensis*	5	Agulhas	9	0	0
*C. agulhensis*	6	Agulhas	11	0	0
*C. agulhensis*	7	Agulhas	2	0	0
*C. sinicus*	1	West Japan	11	6	1
*C. sinicus*	2	West Japan	14	1	2
*C. sinicus*	3	East Japan	23	12	3

Population names are as used in the text and statistical analyses.

DNA from adult females was obtained using the DNeasy® Blood and Tissue Kit (Qiagen) and eluted to a final volume of 200 µL. A 507 base-pair (bp) region of mtCOI was amplified using the consensus primers LCO-1490 and HCO-2198 [54]. PCR reactions were carried out in 25 µL volume, with 3 µL template DNA, 2.5 µL 25 mM MgCL_2_, 1 µL of dNTPs (0.2 mM of each dNTP), 1 µL 10 µM each of forward and reverse primer, 0.75 units GoTaq Flexi DNA polymerase, and 5 µL 5× Green GoTaq Flexi buffer (Promega) and H_2_O to a final volume of 25 µL. Twenty *C. agulhensis* and 7 *C. sinicus* sequences were determined using a pair of universal primers that define the mtCOI barcode region; the sequences were used to design the internal species-specific PCR and sequencing primers: LCO-1576 5′-ATTCGATTAGAGTTAGGTCAAGC-3′ and HCO-2081 5′-CATAAAATGTGGTGTTCAGGTTACG-3′. Use of these primers was necessary to obtain clean sequences from poorly-preserved *C. sinicus* samples. The mtCOI PCR protocol used was: 1 step of 94°C for 3 min; 35 cycles of 94°C for 30 s, 60°C for 45 s, 72°C for 45 s; 1 step of 72°C for 7 min. A 503 bp region of CS was amplified using the primers: CS-9F 5′-ATTCCGTGGGTACACCATCC-3′ and CS-514R 5′-TTGTCAAGTACAGTCTCATCAGC-3′ (Ebru Unal, University of Connecticut, unpubl. data). The CS PCR protocol used was: 1 step of 94°C for 1 min; 35 cycles of 94°C for 20 s, 55°C for 30 s, 72°C for 1 min; 1 step of 72°C for 5 min. A 658 bp region of 28S rRNA was amplified using the primers: 28S-F1a 5′-GCGGAGGAAAAGAAACTAAC-3′ and 28S-R1a 5′- GCATAGTTTCACCATCTTTCGGG-3′ [55]. The 28S rRNA PCR protocol used was: 1 step of 94°C for 4 min; 35 cycles of 94°C for 45 s, 50°C for 40 s, 72°C for 90 s; 1 step of 72°C for 15 min. Successful PCR products were electrophoresed in a 1% agarose gel. Products that showed a strong band of the correct size were selected and cleaned using a QIAquick® PCR purification kit (Qiagen). The PCR primers were also used for sequencing with the BigDye ® Terminator Ver. 3.1 Cycle Sequencing Kit (Applied Biosystems Inc., ABI) and protocols. Sequencing was done on an ABI 3130 Genetic Analyzer 4-capillary automated DNA sequencer. Sequences were edited and aligned using the Molecular Evolutionary Genetics Analysis (MEGA, Ver. 4.0; [56]).

### Data Analysis

The program DnaSP Ver. 5 [57] was used to calculate haplotype diversity (H_d_) and nucleotide diversity (π) for the COI data, and also to test for neutrality. Haplotype diversity was standardized using the program RAREFACT Ver.1.0 [58]. Tajima's D [59] and Fu's F_S_ [60] were used to test for neutrality. The diploid CS sequences were recorded using ambiguity codes to represent sites with double peaks in the chromatogram file; these were interpreted as heterozygous sites. The PHASE analysis implemented in DnaSP Ver. 5 was then used to reconstruct haplotypes from the diploid CS sequences based on a Bayesian statistical model [61], [62].

Analysis of Molecular Variation (AMOVA; [63]) and F_ST_ pairwise distances were calculated for the mtCOI and CS data independently using ARLEQUIN Ver. 3.5 [64]. Our F_ST_ pairwise distances for CS were obtained on both phased and unphased data. For the unphased analysis, ambiguous sites were ignored; the phased analysis considered all sites. Two *a priori* hierarchical groupings were tested for the AMOVA analysis of mtCOI data. Variance among groups (Φ_CT_), among populations within groups (Φ_SC_) and within populations (Φ_ST_) was tested for statistical significance after 100,172 permutations. Also, F_ST_ pairwise distances comparing genetic variation (in nucleotide bases) within and among sub-populations in relation to the entire population were calculated [65]. F_ST_ values, which range from 0 (indicating a panmictic population) to 1 (complete separation), were calculated using models and gamma values assigned by jModelTest [66]: pairwise differences for mtCOI (γ = 2.0), Jukes and Cantor for the unphased CS data (γ = 2.0) and Tamura and Nei for the phased CS data (γ = 0.159). Pairwise distances for mtCOI were also calculated among and between *C. sinicus* and *C. agulhensis* using the Kimura 2-Paramater (K2P; [67]) method in MEGA (γ = 2.0). A K2P analysis was chosen to provide a secondary analysis of genetic variation and to adhere with the barcoding literature in which K2P is the most common metric [47], [48]. AMOVA terms and a parsimony haplotype network diagram for mtCOI was constructed using the program TCS Ver. 2.1 [68]. In the diagram, haplotype frequencies are represented by size and graphics were assigned to represent the four populations.

A Maximum Likelihood tree for the 28S rRNA gene sequences was computed using RAxML Ver. 7.0.3 [69], [70], under the GTRGAMMA option (i.e., GTR model of nucleotide substitution with the Γ model of rate heterogeneity) and a complete random starting tree (option -d) for 1,000 bootstrap replicates. Phasing the data was not necessary, as we did not observe any ambiguous sites. Analysis was done for multiple alignments with additional published sequences obtained from GenBank for *C. helgolandicus* (GenBank Acc. No. HM997038), *C. marshallae* (EF460770), *C. glacialis* (EF460768), *C. hyperboreus* (EF460769), *C. simillimus* (EU914255) and *C. finmarchicus* (EU375491). GenBank sequences for *Neocalanus plumchrus* (AF385471) and *Neocalanus cristatus* (AF385470) were used as outgroups.

## Results

### Cytochrome *c* Oxidase 1

**Figure 2:**
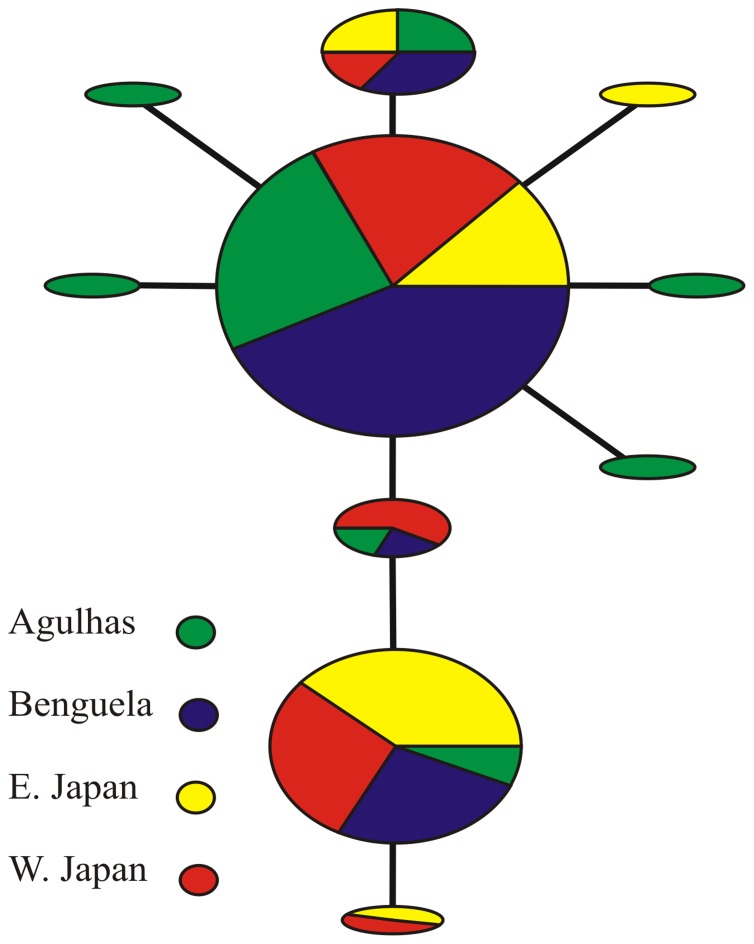
TCS network diagram for mtCOI haplotypes for *C. sinicus* and *C. agulhensis*. Numbers of individuals are reflected in the pie size; pie slices represent the frequencies of each haplotype in that population. See text for explanation.

A total of 48 mtCOI sequences for *C. sinicus* and 46 sequences for *C. agulhensis* were analyzed after trimming the multiple-sequence alignment to a final length of 507 bp (Table 2). For the AMOVA analysis, two *a priori* groups, Japan and South Africa, were established. Each group was further divided into two populations, respectively: East and West Japan, and Benguela and Agulhas. All four populations shared three common haplotypes, with a total of 8 haplotypes for *C. agulhensis* (GenBank Acc. Nos. JF430012 - JF430019) and 6 for *C. sinicus* (JF430039 - JF430044; Fig. 2). There were 5 unique mtCOI haplotypes that occurred within one sample each; these satellite haplotypes each resulted from a single base change (Fig. 2).

The neutrality test using Tajima's D was not significant, suggesting neutral evolution. However, *C. agulhensis* had a negative Fu's F_S_ value that was very significant (−4.60; P = 0.001); *C. sinicus* was neither negative nor significant (0.005, P = 0.53). Haplotype diversity was high and comparable among the groups (Table 3). The high and very similar observed levels of haplotype and nucleotide diversity also suggested the shared genetic composition of the two species; this was discerned based on their shared haplotypes and low to no genetic variation, despite the rapid mutational rate of mtCOI. The pairwise F_ST_ values for mtCOI were low, with the lowest value between the West Japan and Benguela populations. The largest pairwise F_ST_ values for mtCOI were found between East Japan and Agulhas (F_ST_ = 0.228; P = 0.001) and between West Japan and Agulhas (F_ST_ = 0.152; P = 0.010; Table 4). The AMOVA test showed low and non-significant variation among the groups, with significant variation within populations (Table 5). A second test to compare Japan and South Africa was run without separating the groups into populations; the resulting F_ST_ value was low and significant (F_ST_ = 0.083, P = 0.003). The K2P test also showed low levels of variation within each group: *C. agulhensis* had the lower average (0.002, ±0.002) compared to *C. sinicus* (0.003, ±0.002); variation between the two groups was also low (0.003, ±0.002; Table 6).

**Table 3:** Diversity measures of mtCOI sequence variation.

	*C. sinicus*	*C. agulhensis*	Both
H_d_	0.695	0.660	0.698
π	0.003	0.002	0.003
H	6	8	-

H_d_ – haplotype diversity , π – nucleotide diversity and H – number of haplotypes.

**Table 4:** F_ST_ distances between populations of *C. sinicus* (West and East Japan) and *C. agulhensis* (Benguela and Agulhas) based on mtCOI.

	West Japan	East Japan	Benguela
East Japan	0.016 n.s.		
Benguela	0.001 n.s.	0.057 n.s.	
Agulhas	0.152*	0.228**	0.036 n.s.

* P - value<0.01,

** P - value<0.005.

**Table 5:** AMOVA analysis of mtCOI variation.

Source of	d.f.	Sum of	Variance	Percentage	Significance
Variation		Squares	Components	of Variation	
Among	1	4.150	0.07333	10.72	n.s.
groups Φ_CT_					
Among	2	1.409	0.00417	0.61	n.s.
populations within					
groups Φ_SC_					
Within	90	54.601	0.60668	88.67	P = 0.01
populations Φ_STd_					
Total	93	60.160	0.68417		

The mtCOI dataset was divided into two groups, South Africa (*C. agulhensis*) and Japan (*C. sinicus*), with each group partitioned into two populations: Benguela and Agulhas, and East Japan and West Japan, respectively. Statistical significance was evaluated based on 100,172 permutations.

**Table 6:** K2P distances between and among *C. sinicus* and *C. agulhensis* based on mtCOI.

	Between	*C. sinicus*	*C. agulhensis*
AVG	0.003	0.003	0.002
MIN	0.000	0.000	0.000
MAX	0.008	0.008	0.006
STDEV	0.002	0.002	0.002

AVG = average, MIN = minimum value, MAX = maximum value; STDEV = standard deviation.

### Citrate Synthase

The CS nucleotide sequences were trimmed to a length of 457 bp for analysis. A total of 19 different sequence phenotypes, four of which are shared, were found for *C. agulhensis* (GenBank Acc. Nos. JF430020 - JF430038), and 16 for *C. sinicus* (JF430045 - JF430060; Table 2). Arlequin Ver. 3.5 was used to calculate F_ST_ values between two groups, Japan and South Africa. The groups were not further subdivided into populations, due to a lack of analyzed samples for all four populations. Tajima's D was not significant for the comparison, but *C. agulhensis* had a negative and highly significant Fu's F_S_ test (−22.82, P<0.0001); *C. sinicus* was also negative and significant (−7.29, P = 0.008). The F_ST_ values were low and significant (F_ST_ = 0.05, P = 0.021) for the unphased diploid data, but not significant for the phased data (F_ST_ = 0.048, P = 0.069).

### Large subunit (28S) rRNA

**Figure 3:**
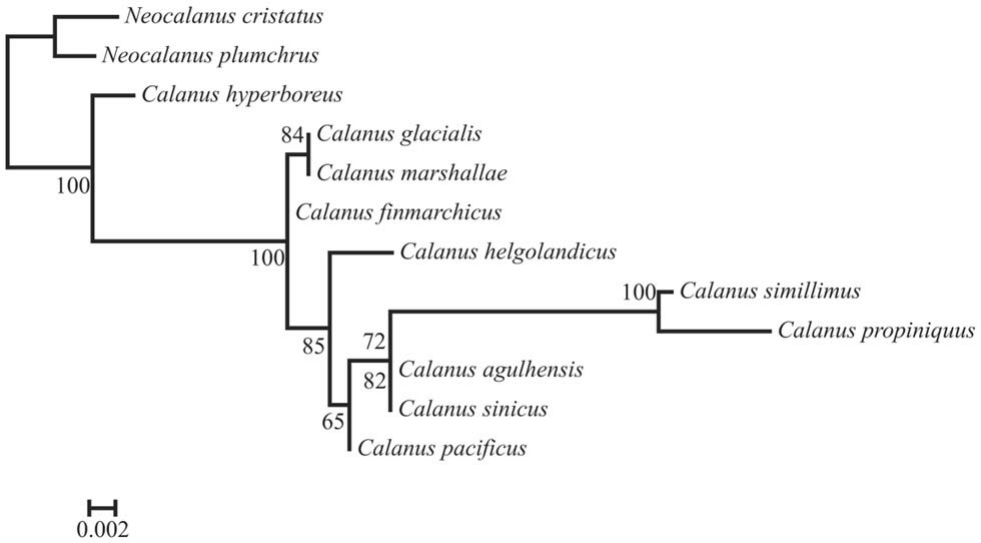
Maximum Likelihood tree for 10 species of *Calanus*. Tree is based on a 658 bp region of the 28S rRNA gene under GTR. Numbers at nodes indicate percentage of recovery after 1,000 bootstraps.

Sequences were determined for the same region of 28S rRNA for *C. agulhensis* (GenBank Acc. No. JF703102), *C. sinicus* (JF703103), *C. propinquus* (JF703105), and *C. pacificus* (JF703104). No nucleotide variation was observed for a 658 bp region among six 28S rRNA sequences for each of *C. agulhensis* and *C. sinicus* (Table 2). There was sufficient genetic variation among most species to allow resolution of relationships within this genus, but no variation between the two species in question (Fig. 3).

## Discussion

The absence of accurate and detailed descriptions of species can complicate the classification needed to adequately study and conserve marine species diversity. Closely related species that lack diagnostic morphological characters, yet share other traits (e.g., behavior, life history, and geographical distribution), present persistent challenges for taxonomists and ecologists. This is further complicated in the marine environment, where clear cut physical characters may be altered in collection; mating behavior and chemical signaling cannot be observed; species may inhabit variable ranges with disjunct populations; cryptic species may have overlapping ranges; and dynamic currents and human intervention may transport species into foreign ecosystems.

This study analyzed three genes to provide new genetic data for re-evaluation of the taxonomic distinctiveness of two sibling species of the copepod genus, *Calanus*. The results confirm very low levels of genetic variation between *C. agulhensis* and *C. sinicus*. The lack of divergence of these genes is not typical among other *Calanus* species.

Although the mtCOI barcode region has been shown to be a reliable molecular character for recognition and discrimination of metazoans [47], including marine metazoans [49], this gene region did not provide evidence of species distinction between *C. agulhensis* and *C. sinicus*. The high and very similar observed levels of haplotype and nucleotide diversity also suggested the shared genetic composition of the two species. The majority of mtCOI variance was found within the sampled populations (i.e., Agulhas, Benguela, etc.) and not among the designated groups (i.e., Japan and South Africa; Table 5). In fact, F_ST_ values similar to those found here are not unusual between geographically isolated, conspecific populations that span vast distances and occupy discontinuous ranges [71], [72], [73]. Similar patterns were observed based on analysis of K2P values of the mtCOI gene and the diploid CS gene (using unphased data), for which low levels of differentiation were found between the two species. We were unable to adequately measure interbreeding or hybridization from the CS data because of the lack of variation observed between the two species

There were no differences between *C. sinicus* and *C. agulhensis* for the 28S rRNA gene region sequenced. This gene shows sufficient variation to resolve most other species of *Calanus*, except for the sibling species *C. marshallae* and *C. glacialis*, and does not resolve the two groups of sibling species within the genus. Although 28S rRNA is not a conclusive diagnostic marker for such closely related species, the gene shows useful rate constancy to evaluate taxonomic relationships at higher taxonomic levels [37].

Overall, these patterns indicate a degree of geographic structure and reproductive isolation that is more characteristic of differences between populations of the same species than between different species. Several considerations necessitate caution in the strength of our conclusions. First is our moderate sample sizes and limited geographic scope of sampling across each species' range. Second, F-statistics alone are insufficient metrics for delimiting species and should be used with caution for taxonomic studies.

Several scenarios based on patterns and pathways of large-scale dispersal or migration may be useful to explain our findings of genetic cohesiveness between *C. sinicus* and *C. agulhensis*. One possibility is that migrants may survive the extensive journey via warm-water surface currents that flow through the Indian Ocean and around Africa into the South Atlantic [74], where they may contribute to and establish populations around South Africa. The South Equatorial current flows west toward Africa from the Indonesian seas and then south into the Mozambique Channel, combining with the Agulhas Current and traveling toward the South Atlantic. The prominent Agulhas Current and Return Current also provide a convenient loop that could deposit migrants on the western and northern region of the Agulhas Bank, where recruits would find optimal living conditions [75]. Transport may well have been intermittent or episodic, since these major pathways are strongly influenced by climatic conditions and other factors [74], [76]. To our knowledge, neither species has been documented or observed in the Indian Ocean; it should be noted that the proposed current system flows substantially south of any suitable coastal habitats.

Another possible transport pathway is via ships' ballast water; *C. sinicus* was reported in 31 bulk cargo carriers traveling from Japanese ports to Australia [77]. In the Russian port of Vladivostok, *C. sinicus* was found in samples of ballast water coming from the port of Longkou [78]; the species had been previously unrecorded in the Peter the Great Bay. Nuwer [9] noted ballast water transport as a possibility, noting long-distance transport of the copepod *Eurytemora americana*, which was introduced to Argentina from the Northern Hemisphere. Our genetic data are consistent with such a scenario: the very significant Fu's F_S_ value (−22.82; P<0.001) for *C. agulhensis* is consistent with a recent population expansion. Also, Robinson et al. [79] note that there is little investigation into invasive species of South Africa, especially near the Indian Ocean, citing a lack of full-time professional marine taxonomists.

Feeding dynamics and life history may account for the establishment and dominance of this copepod. The center of distribution for *C. algulhensis* is the Agulhas Bank, which has a high concentration of small phytoplankton [25] and may be a suboptimal habitat for older stages. The species' progression from east to west during their ontogenetic maturation provides access to stage-specific preferred food and nutrient conditions, and has allowed them to prosper and retain a healthy population. [17]. Maintaining a home range south of the Benguela Current may allow *C. agulhensis* to prosper without competing with *Calanoides carinatus* and other copepods that inhabit the area. Hugget et al. [80] proposed a model whereby the European anchovy, *Engraulis encrasicolus*, replaced a South African population that became extinct; they suggested this was possible since *E. encrasicolus* spawns in the warm Agulhas Bank waters, where their larvae avoid lethal temperatures, with a westward progression of life stages following a similar path to that of *C. agulhensis*. Investigation of this hypothesis is problematical, because of a lack of fossil records and specimens collected before 1960.

Finally, it is possible that *C. agulhensis* and *C. sinicus* are part of a cryptic species complex that exists as two – or more – geographically isolated species with indistinguishable genetics (e.g., [2]) In this scenario, the observed genetic similarities could be attributed to plesiomorphic haplotypes (i.e., alleles inherited from a common ancestor). The defining biological characteristics may be unobservable, including chemical recognition during mating and reproduction, fertilization barriers, etc. and/or differences in reproductive behavior and synchronicity may be caused by geographic isolation [3].

Overall, our analysis of mtCOI, CS, and 28S rRNA variation within and among the analyzed samples of *C. sinicus* and *C. agulhensis* consistently showed low to zero levels of genetic divergence between the species. The great majority of molecular variation was observed within the sampled populations, rather than between the species, suggesting that we have sampled a large panmictic population that spans distinct ocean basins. Further, our results concur with earlier studies, such as that by Nuwer [9], which have questioned whether *C. agulhensis* warrants status as a distinct species. Explaining this result and exploring the underlying mechanisms that link these two populations separated by large geographic distances and continental barriers will require further investigation of the species' ecology, behavior, life history, morphology, physiology, and – not least – molecular genetics.

## References

[1] Knowlton N (1993) Sibling species in the sea. Annu Rev Ecol Syst 24: 189–216.

[2] Knowlton N (2000) Molecular genetic analyses of species boundaries in the sea. Hydrobiologia 420: 73–90.

[3] Bickford D, Lohman DJ, Sodhi NS, Ng PKL, Meier R, et al (2007) Cryptic species as a window on diversity and conservation. Trends Ecol Evol 22: 148–155. 17129636 10.1016/j.tree.2006.11.004

[4] De Vargas C, Norris R, Zaninetti L, Gibb SW, Pawlowski J (1999) Molecular evidence of cryptic speciation in planktonic foraminifers and their relation to oceanic provinces. Proc Natl Acad Sci 96: 2864–2868. 10077602 10.1073/pnas.96.6.2864 PMC15860

[5] Suatoni E, Vicario S, Rice S, Snell T, Caccone A (2006) An analysis of species boundaries and biogeographic patterns in a cryptic species complex: The rotifer - *Brachionus plicatilis* . Mol Phylogenet Evol 41: 86–98. 16815046 10.1016/j.ympev.2006.04.025

[6] Goetze E (2003) Cryptic speciation on the high seas; global phylogenetics of the copepod family Eucalanidae. Proc R Soc B-Biol Sci 270: 2321–2331. 10.1098/rspb.2003.2505 PMC1691510 14667347

[7] Adamowicz SJ, Menu-Marque S, Hebert PDN, Purvis A (2007) Molecular systematics and patterns of morphological evolution in the Centropagidae (Copepoda: Calanoida) of Argentina. Biol J Linn Soc 90: 279–292.

[8] Durbin A, Hebert PDN, Cristescu MEA (2008) Comparative phylogeography of marine cladocerans. Mar Biol 155: 1–10.

[9] Nuwer M (2008) Genetic structure and speciation in planktonic copepods: global phylogeography of the *Calanus helgolandicus* clade. Dissertation, University of Washington, Washington.

[10] Bradford JM (1988) Review of the taxonomy of the Calanidae (Copepoda) and the limits to the genus *Calanus* . Hydrobiologia 167/168: 73–81.

[11] Bradford-Grieve JM, Ahyong ST (2010) Phylogenetic relationships among genera in the Calanidae (Crustacea: Copepoda) based on morphology. J Nat Hist 44: 279–299.

[12] Brodsky KA (1972) Phylogeny of the family Calanidae (Copepoda) on the basis of a comparative morphological analysis of its characters. Issled Fauny Morei 12: 1–127 (English translation, Israel Program for Science Translations 1975).

[13] Frost B (1971) Taxonomic status of *Calanus finmarchicus* and *C. glacialis* (Copepoda), with special reference to adult males. J Fish Res Board Can 28: 23–30.

[14] Frost B (1974) *Calanus Marshallae*, a new species of Calanoid Copepod closely allied to the sibling species *C. finmarchicus* and *C. glacialis* . Mar Biol 26: 77–99.

[15] Cleve PT (1904) Plankton of the South Africa seas. 1. Copepoda. Mar Invest S Afr 3: 177–210.

[16] De Decker AHB, Marska G, Kaczmaruk BZ (1991) A new species of *Calanus* (Copepoda, Calanoida) from South African waters. Ann S Afr Mus 101: 27–44.

[17] Huggett J, Richardson AJ (2000) A review of the biology and ecology of *Calanus Agulhensis* off South Africa. ICES J Mar Sci 57: 1834–1849.

[18] Richardson AJ, Field JG, Fowler JL, Mitchell-Innes BA, Verheye HM (2003) Seasonal and event-scale variation in growth of *Calanus agulhensis* (Copepoda) in the Benguela upwelling system and implications for spawning of sardine *Sardinops sagax* . Mar Ecol Prog Ser 254: 239–251.

[19] Brodsky KA (1965) Variability and systematics of the species of the genus Calanus (Copepoda). 1. *Calanus pacificus* Brodsky, 1948 and *C. sinicus* Brodsky, sp. n. Issle Fauny Morei 3: 22–71.

[20] Hulsemann K (1994) *Calanus sinicus* Brodsky and *C. jashnovi* nom. nov. (Copepoda: Calanoida) of the North-West Pacific Ocean: a comparison, with notes on the integumental pore pattern in *Calanus* s. str. Invertebr Tax 8: 1461–1482.

[21] Uye S, Huang C, Onbe T (1990) Ontogenetic diel vertical migration of the planktonic copepod *Calanus sinicus* in the Inland Sea of Japan. Mar Biol 104: 389–396.

[22] Uye S (2000) Why does *Calanus sinicus* prosper in the shelf ecosystem of the Northwest Pacific Ocean? ICES J Mar Sci 57: 1850–1855.

[23] Huang C, Onbe T, Uye S (1993) Geographic distribution, seasonal life cycle, biomass and production of a planktonic copepod *Calanus sinicus* in the Inland Sea of Japan and its neighboring Pacific Ocean. J Plankton Res 15: 1229–1246.

[24] Uye S, Murase A (1997) Relationship of egg production rates of the planktonic copepod *Calanus sinicus* to phytoplankton availability in the Inland Sea of Japan. Plankton Biol Ecol 44: 3–11.

[25] Verheye HM, Carter RA, Huggett J, Hutchings L, Painting SJ, et al (1994) Community structure, distribution and trophic ecology of zooplankton on the Agulhas Bank with special reference to copepods. S Afr J Sci 90: 164–165.

[26] Probyn TA, Brown PC, Carter RA, Hutchings L, Mitchell-Innes BA (1994) A review of primary production and related processes on the Agulhas Bank. S Afri J Sci 90: 166–173.

[27] Peterson W (1998) Life cycle strategies of copepods in coastal upwelling zones. J Mar Syst 15: 313–326.

[28] Takahashi M, Fujita Y, Furuya K, Hattori A, Ishimaru T, et al (1980) Upwelling plumes in Sagami Bay and adjacent water around the Izu Islands, Japan. J Ocean Soc Jap 36: 209–216.

[29] Atkinson LP, Blanton JO, Lee TN, Ishimaru T, Ishizaka J, et al (1987) Observations of upwelling around the Izu Peninsula, Japan: May 1982. J Ocean Soc Jap 4: 389–103.

[30] Bucklin A, Frost BW, Kocher TD (1992) DNA sequence variation of the mitochondrial 16S rRNA in *Calanus* (Copepoda: Calanoida): intraspecific and interspecific patterns. Mol Mar Biol Biotechnol 1: 397–407.

[31] Bucklin A, Frost BW, Kocher TD (1995) Molecular systematics of six *Calanus* and three *Metridia* species (Calanoida: Copepoda). Mar Biol 121: 655–664.

[32] Hill RS, Allen LD, Bucklin A (2001) Multiplexed species-specific PCR protocol to discriminate four N. Atlantic *Calanus* species, with an mtCOI gene tree for ten *Calanus* species. Mar Biol 139: 279–287.

[33] Unal E, Frost B, Kideys A, Armbrust V (2006) Phylogeography of *Calanus helgolandicus* and the Black Sea copepod *Calanus euxinus*, with notes on *Pseudocalanus elongatus* (Copepoda, Calanoida). Deep Sea Res Part II 53: 1961–1975.

[34] Fleminger A, Hulsemann K (1987) Geographical variation in *Calanus helgolandicus* s.l. (Copepoda, Calanoida) and evidence of recent speciation of the Black Sea Population. Biol Oceanogr 5: 43–81.

[35] Bucklin A, Kocher TD (1996) Source regions for recruitment of *Calanus finmarchicus* to Georges Bank: evidence from molecular population genetic analysis of mtDNA. Deep Sea Res Part II 43: 1665–1681.

[36] Bucklin A, Bently AM, Franzen SP (1998) Distribution and relative abundance of *Pseudocalanus moultoni* and *P. newmani* (Copepoda: Calanoida) on Georges Bank using molecular identification of sibling species. Mar Biol 132: 97–106.

[37] Braga E, Meyer A, Yen J, Zardoya R (1999) Mitochondrial and nuclear rRNA based copepod phylogeny with emphasis on the Euchaetidae (Calanoida). Mar Biol 133: 79–90.

[38] Taniguchi M, Christen R, Kanehisa T, Sawabe T (2004) Molecular phylogeny of *Neocalanus* copepods in the subarctic Pacific Ocean, with notes on non-geographical genetic variations for *Neocalanus cristatus* . J Plankton Res 26: 1249–1255.

[39] Machida RJ, Miya MU, Nishida M, Nishida S (2006) Molecular phylogeny and evolution of the pelagic copepod genus *Neocalanus* (Crustacea: Copepoda). PLoS Biol 3: 1071–1079.

[40] Thum RA, Harrison RG (2009) Deep genetic divergences among morphologically similar and parapatric *Skistodiaptomus* (Copepoda: Calanoida: Diaptomidae) challenge the hypothesis of Pleistocene speciation. Biol J Linn Soc 96: 150–165.

[41] Bucklin A, Frost BW, Bradford-Grieve J, Allen LD, Copley NJ (2003) Molecular systematic and phylogenetic assessment of 34 calanoid copepod species of the Calanidae and Clausocalanidae. Mar Biol 142: 333–343.

[42] Lindeque PK, Harris RP, Jones MB, Smerdon GR (1999) Simple molecular method to distinguish the identity of *Calanus* species (Copepoda: Calanoida) at any developmental stage. Mar Biol 133: 91–96.

[43] Yebra L, Bonnet D, Harris RP, Lindeque PK, Peijnenburg KTCA (2011) Barriers in the pelagic: population structuring of *Calanus helgolandicus* and *C. euxinus* in European waters. Mar Ecol Prog Ser 428: 135–149.

[44] Feng D, Shou-Hslen L, Xiao-un Y (2010) Molecular systematics and diversification of the Asian scimitar babblers (Timaliidae, Aves) based on mitochondrial and nuclear DNA sequences. Mol Phylogenet Evol 57: 1268–1275. 20937399 10.1016/j.ympev.2010.09.023

[45] Pereira TJ, Fonseca G, Mundo-Ocampo M, Guilherme BC, Rocha-Olivares A (2010) Diversity of free-living marine nematodes (Enoplida) from Baja California assessed by integrative taxonomy. Mar Biol 157: 1665–1678. 24391248 10.1007/s00227-010-1439-z PMC3873033

[46] Unal E, Bucklin A (2010) Basin-scale population genetic structure of the planktonic copepod *Calanus finmarchicus* in the North Atlantic Ocean. Prog Oceanogr 87: 175–185.

[47] Hebert PD, Cywinska A, DeWaard JR, Ball SL (2003) Biological identification through DNA barcodes. Proc R Soc B-Biol Sci 270: 313–321. 10.1098/rspb.2002.2218 PMC1691236 12614582

[48] Meyer CP, Paulay G (2005) DNA barcoding: error rates based on comprehensive sampling. Mar Biol 148: 2229–2238. 10.1371/journal.pbio.0030422 PMC1287506 16336051

[49] Bucklin A, Steinke D, Blanco-Bercial L (2011) DNA Barcoding of Marine Metazoans. Annu Rev Mar Sci 3: 471–508. 10.1146/annurev-marine-120308-080950 21329214

[50] Nuwer M, Frost B, Ambrust EV (2008) Population structure of the planktonic copepod *Calanus pacificus* in the North Pacific Ocean. Mar Biol 156: 107–115.

[51] Lin YS, Fang LP, Cao WQ, Li SJ (2005) Mitochodrial DNA COI sequence analysis of *Calanus sinicus* (Copepod) in Qingdao Waters. J Xiamen University (Natural Science) 2005-1

[52] Ragionieri L, Fratini S, Schubart CD, Vannini M (2009) Phylogenetic and morphometric differentiation reveal geographic radiation and pseudo-cryptic speciation in a mangrove crab from the Indo-West Pacific. Mol Phylogenet Evol 52: 825–834. 19394431 10.1016/j.ympev.2009.04.008

[53] Shih HT, Chen GX, Chien IC, Ng PKL, Zhou XM (2010) Recent vicariant and dispersal events affecting of the phylogeny and biogeography of East Asian freshwater crab genus *Nanhaipotamon* (Decapoda: Potamidae). Mol Phylogenet Evol 58: 427–438. 21095233 10.1016/j.ympev.2010.11.013

[54] Folmer O, Black M, Hoeh W, Lutz W, Vrijenhock R (1994) DNA primers for amplification of mitochondrial cytochrome *c* oxidase subunit I from diverse metazoan invertebrates. Mol Mar Biol Biotechnol 3: 294–299. 7881515

[55] Ortman BD (2008) DNA barcoding the Medusozoa and Ctenophora. Dissertation, University of Connecticut, Connecticut.

[56] Tamura K, Peterson D, Peterson N, Stecher G, Nei M, et al (2011) MEGA5: Molecular Evolutionary Genetics Analysis using Maximum Likelihood, Evolutionary Distance, and Maximum Parsimony Methods. Mol Biol and Evol 28: 2731–2739. 21546353 10.1093/molbev/msr121 PMC3203626

[57] Librado P, Rozas J (2009) DnaSP v5: a software for comprehensive analysis of DNA polymorphism data. Bioinformatics 25: 1451–1452. 19346325 10.1093/bioinformatics/btp187

[58] Petit R, El Mousadik A, Pons O (1998) Identifying populations for conservation on the basis of genetic markers. Conserv Biol 12: 844–855.

[59] Tajima F (1989) Statistical method for testing the neutral mutation hypothesis by DNA polymorphism. Genetics 123: 585–595. 2513255 10.1093/genetics/123.3.585 PMC1203831

[60] Fu YX (1997) Statistical tests of neutrality against population growth, hitchhiking and background selection. Genetics 147: 915–925. 9335623 10.1093/genetics/147.2.915 PMC1208208

[61] Stephens M, Donnelly P, Smith NJ (2001) A new statistical method for haplotype reconstruction from population data. Amer J Hum Genet 68: 978–989. 11254454 10.1086/319501 PMC1275651

[62] Stephens M, Donnelly P (2003) A comparison of bayesian methods for haplotype reconstruction from population genotype data. Amer J Hum Genet 73: 1162–1169. 14574645 10.1086/379378 PMC1180495

[63] Excoffier L, Smouse PE, Quattro JM (1992) Analysis of Molecular Variance inferred from metric distances among DNA haplotypes: application to human mitochondrial DNA restriction data. Genetics 131: 479–491. 1644282 10.1093/genetics/131.2.479 PMC1205020

[64] Excoffier L, Lischer HEL (2010) Arlequin suite ver 3.5: A new series of programs to perform population genetic analyses under Linux and Windows. Mol Ecol Res 10: 564–567. 10.1111/j.1755-0998.2010.02847.x 21565059

[65] Holsinger KE, Weir BS (2009) Genetics in geographically structured populations: defining, estimating and interpreting FST. Nat Rev Genet 10: 639–50. 19687804 10.1038/nrg2611 PMC4687486

[66] Posada D (2008) jModelTest: Phylogenetic model averaging. Mol Biol Evol 25: 1253–1256. 18397919 10.1093/molbev/msn083

[67] Kimura M (1980) A simple method for estimating evolutionary rate of base substitutions through comparative studies of nucleotide sequences. J Mol Evol 16: 111–120. 7463489 10.1007/BF01731581

[68] Clement M, Crandall K, Posada D (2000) TCS: a computer program to estimate gene genealogies. Mol Ecol 9: 1657–1660. 11050560 10.1046/j.1365-294x.2000.01020.x

[69] Stamatakis A (2006) RAxML-VI-HPC: maximum likelihood-based phylogenetic analyses with thousands of taxa and mixed models. Bioinformatics 22: 2688–2690. 16928733 10.1093/bioinformatics/btl446

[70] Stamatakis A, Blagojevic F, Nikolopoulos D, Antonopoulos C (2007) Exploring new search algorithms and hardware for phylogenetics: RAxML meets the IBM cell. J VLSI Signal Process 48: 271–286.

[71] Goetze E (2005) Global population genetic structure and biogeography of the oceanic copepods *Eucalanus hyalinus* and *E. spinifer* . Evolution 59: 2378–2398. 16396179

[72] Eberl R, Cohen S, Cipriano F, Carpenter EJ (2007) Genetic diversity of the pelagic harpacticoid copepod *Macrosetella gracilis* on colonies of the cyanobacterium *Trichodesmium* spp. Aqua Biol 1: 33–43.

[73] Blanco-Bercial L, Álvarez-Marqués F, Bucklin A (2011) Comparative phylogeography and connectivity of sibling species of the marine copepod *Clausocalanus* (Calanoida). J Exp Mar Biol Ecol 404: 108–115.

[74] Steele JH, Thorpe SA, Turekian KK (2009) Ocean Currents: A derivative of the encyclopedia of Ocean Sciences. Academic Press, London, pp 125–13.

[75] Hall C, Lutjeharms JRE (2011) Cyclonic eddies identified in the Cape Basin of the South Atlantic Ocean. J Mar Syst 85: 1–10.

[76] Gordon AL (1986) Interocean exchange of thermocline water. J Geophys Res 91: 505046.

[77] Williams RJ, Griffiths FB, Van der Wal EJ, Kelly J (1988) Cargo vessel ballast water as a vector for the transport of non-indigenous marine species. Estuar Coast Shelf Sci 26: 409–420.

[78] Kasyan VV (2010) Holoplankton of ship ballast water in the port of Vladivostok. Russ J Mar Sci 36: 167–175.

[79] Robinson TB, Griffiths CL, McQuaid CD, Rius M (2005) Marine alien species of South Africa - status and impacts. Afr J Mar Sci 27: 297–306.

[80] Huggett J, Feron P, Mullon C, Pierrick P (2003) Modelling the transport success of anchovy *Engraulis encrasicolus* eggs and larvae in the southern Benguela: the effect of spatio-temporal spawning patterns. Mar Ecol Prog Ser 250: 247–262.

